# Hepatocellular Carcinoma: Special Issue Highlights

**DOI:** 10.3390/cancers12082026

**Published:** 2020-07-24

**Authors:** Medhavi Gupta, Renuka V. Iyer

**Affiliations:** 1 Department of Medicine, Roswell Park Comprehensive Cancer Center, Buffalo, NY 14263, USA; medhavi.gupta@roswellpark.org; 2 Division of Gastrointestinal Medicine, Department of Medicine, Roswell Park Comprehensive Cancer Center, Scott Bieler Clinical Sciences Center, Buffalo, NY 14263, USA

Hepatocellular carcinoma (HCC) is the second leading cause of cancer-related mortality worldwide. Sorafenib remained the main stay treatment for patients with advanced HCC for over a decade [1,2]. In the last four years, six new systemic therapies have shown efficacy in randomized controlled trials and increased options for those with advanced disease [3,4,5,6,7,8]. Despite these advances, cures are rare and survival short. Efforts focused on identifying novel targets and combination strategies and optimizing patient selection through biomarker development are still needed. This issue features a series of articles focusing on the molecular pathogenesis of HCC, novel therapeutic and biomarker development strategies and the impact of current therapies on quality of life (QoL).

A minority of patients have locoregional disease at the time of presentation and are candidates for potentially curative options. Surgical resection remains one of the cornerstone curative strategies but, given the underlying liver disease, is not an option for many. Ju et al. review the advantages and disadvantages of anatomical resection (AR) over non-anatomical resection (NAR) in HCC patients and outline outcome measures of both the techniques [9]. AR can potentially reduce micrometastatic disease related recurrence rates, due to the ligation of portal venous flow proximal to the tumor. However, there is inconclusive data regarding the use of AR in cirrhotic patients, warranting a randomized controlled trial in this group of patients. Liver directed therapies (LDT) in appropriate candidates can be curative, or offer durable control of local disease and palliation when needed. Viveiros et al. review the role of different LDT modalities and discuss the strategies of combining LDTs with systemic therapies [10]. Kok et al. report a large nationwide cohort study in Taiwan evaluating the role of adding transarterial chemoembolization (TACE) to sorafenib among propensity-score-matched patients with advanced disease and Child-Pugh A (CPA) liver function [11]. The combination had a superior survival, with a 26% reduction in mortality and a comparable toxicity profile. In a retrospective study, Guiu et al. report the role of TACE with idarubicin-loaded tandem drug-eluting beads in unresectable HCC patients. The regimen showed a favorable efficacy with a favorable overall response rate (65%), OS (35 months) and time-to-treatment failure (14 months), warranting further testing in prospective studies [12]. Dubbelboer explore the synergistic effects of doxorubicin and hypoxia in liver cancer cell lines [13]. Different cell lines had varied responses to hypoxia and differed in oncogenic protein profiles, indicating intra and inter-tumor heterogeneity.

Limited models exist for studies of prevention and rationally done studies with tolerable therapies, when given long term, are much needed to design chemoprevention trials. In the woodchuck model of hepatitis B related hepatocellular cancer, Iyer et al. demonstrate the chemo prevention potential, tolerability and immunomodulatory role of low dose sorafenib. High doses of sorafenib upregulated nuclear factor of activated T cells 1 (NFAT1) signaling, and were associated with an immunosuppressive phenotype, with reduced T cell proliferation and enhanced PD-1 expression [14]. Dose related immunosuppressive effects of sorafenib underscore the importance of careful dose selection for the optimal designing of future clinical trials.

This issue contains four original research reports examining novel therapies in preclinical models (n = 3) and patients (n = 1). Chen et al. show that magnolol, a compound extracted from the bark of Magnolia officinalis, enhances the therapeutic efficacy of sorafenib via protein kinase B (AKT) inactivation [15]. Co-administration of LY294002 (AKT inhibitor) with sorafenib increased sorafenib-induced cytotoxicity, the increased expression of anti-apoptotic proteins and the induction of extrinsic/intrinsic apoptotic pathways. The authors suggest a novel treatment strategy involving a combination of sorafenib with magnolol. Therapeutic potential of another natural extract, fisetin, is explored in a study by Liu et al. [16]. In this report, authors demonstrate that protein phosphatase 1 (PP1) is highly expressed in histone deacetylase inhibitors-resistant (HDACis-R) cells and treatment with fisetin reduced PP1 expression, upregulated phosphorylated eukaryotic translation initiation factor 2 subunit α and induced apoptosis. Fisetin could be potentially exploited for HDACis-R tumors. Modica et al. evaluated the role of calcium modulation in epidermal growth factor receptor (EGFR) wild type HCC cell lines [17]. Their work shows that calcium is a key regulator of cell cycle proliferation or apoptosis and calcium chelation is associated with EGFR downregulation. Calcium chelators, along with EGFR inhibitors, could be exploited to lower the doses, and potentially overcome resistance of EGFR-TKIs. Mahalingam et al. report the results of a single-arm phase II study, evaluating the role of a prostate-specific membrane antigen (PSMA) targeted prodrug, mipsagargin (G-202) in the second-line setting after sorafenib for advanced HCC pts [18]. In this study, G-202 was relatively well tolerated, resulted in disease stabilization and was associated with reduced blood flow in lesions, warranting a larger clinical study.

Several papers in the issue comprehensively review different therapies under development. Jiang et al. review the role of AMP-activated protein kinase (AMPK) in HCC [19]. Downregulation of AMPK in HCC is associated with tumorigenesis, cell cycle progression, survival and invasion. Authors discuss the anti-HCC role of several AMPK activators, including metformin. Nishida et al. review the role of an oncofetal glycoprotein, glypican-3 (GPC3) in HCC progression and summarize the latest GPC3-targeting strategies [20]. Reghupaty et al. review the landscape of gene therapy in HCC, highlighting various targeted genes and delivery systems [21]. Mahipal et al. review the evolution of cytokines, vaccines, cellular therapies and immune check point inhibitors, and discuss the future directions of immunotherapies in HCC [22]. 

The lack of or limited available tumor tissue in hepatocellular cancer patients poses a challenge in biomarker development. To this end, various groups have suggested using readily available non-invasive laboratory values, to aid patient selection and prognostication. Ueshima et al., demonstrate the effect of baseline liver function in terms of the Child-Pugh score and ALBI (albumin-bilirubin) grade on efficacy and adverse event outcomes of patients treated with lenvatinib [23]. Antkowiak et al. compare the prognostic ability of ALBI in comparison to the Child-Pugh score in patients treated with yttrium-90 radioembolization [24]. Chon et al. investigated the predictive ability of the neutrophil-to-lymphocyte ratio (NLR) in patients undergoing TACE and developed a 14-point predictive model [25]. Pavlovic et al. provide a comprehensive review of the role of platelets on pathogenesis in HCC and discuss their impact on tumor cells and the tumor microenvironment [26].

The plethora of treatment modalities available to manage HCC patients, many showing efficacy compared to placebo but not compared head to head, present unique challenges in patient selection and sequencing. Standardized approaches are lacking, due to the differences in biological characteristics, patient comorbidities and the paucity of data regarding the appropriate sequencing of novel therapies. In a review by Gholam et al., the authors highlight the importance of multidisciplinary teams to maximize the outcomes of HCC patients, especially in the setting of unresectable disease, and the important roles various subspecialists will play to ensure optimal patient outcomes [27]. 

In rapidly fatal cancers like HCC, the goal of therapy is an improvement in the quality of life (QoL), in addition to prolonging survival. This has been challenging in the HCC space, due to multiple factors, including absent or lack of standardized quality of life reporting in clinical trials, as well as worse liver function and performance status of real-world patients. Zhou et al. summarize the efficacy and safety outcomes of systemic treatment options, along with QoL data, and provide a reference for management of treatment toxicities [28]. Li et al. review the impact of the current FDA approved systemic therapies on QoL parameters and suggest that a better understanding of QoL outcomes could guide appropriate sequencing strategies of novel therapies [29]. 

In conclusion, despite the recent additions to our armamentarium of drugs, HCC remains an area with unmet need. Although more therapy options exist than before, determination of optimal sequencing of therapies, combinations and the impact of these decisions on patient-reported QoL, and on cost of overall care delivery are new challenges that we face. This Special Issue illuminates further understanding into the biology of this aggressive disease and provides innovative solutions for development of new therapies and biomarkers, as our collective efforts towards improving the survival and quality of life outcomes of HCC patients continue. 
